# Translational regulation of cell invasion through extracellular matrix—an emerging role for ribosomes

**DOI:** 10.12688/f1000research.143519.1

**Published:** 2023-11-29

**Authors:** David R. Sherwood, Isabel W. Kenny-Ganzert, Siddharthan Balachandar Thendral

**Affiliations:** 1 Biology, Duke University, Durham, North Carolina, 27708, USA

**Keywords:** cell invasion, cell migration, translational regulation, translation initiation, ribosomes, ribosome biogenesis, ribosome localization

## Abstract

Many developmental and physiological processes require cells to invade and migrate through extracellular matrix barriers. This specialized cellular behavior is also misregulated in many diseases, such as immune disorders and cancer. Cell invasive activity is driven by pro-invasive transcriptional networks that activate the expression of genes encoding numerous different proteins that expand and regulate the cytoskeleton, endomembrane system, cell adhesion, signaling pathways, and metabolic networks. While detailed mechanistic studies have uncovered crucial insights into pro-invasive transcriptional networks and the distinct cell biological attributes of invasive cells, less is known about how invasive cells modulate mRNA translation to meet the robust, dynamic, and unique protein production needs of cell invasion. In this review we outline known modes of translation regulation promoting cell invasion and focus on recent studies revealing elegant mechanisms that expand ribosome biogenesis within invasive cells to meet the increased protein production requirements to invade and migrate through extracellular matrix barriers.

**Abbreviations**
aa-tRNA: Aminoacyl-tRNA
ECM: Extracellular matrix
EMT: Epithelial-mesenchymal transition
ER: Endoplasmic reticulum
IRES: Internal ribosome entry site
Met-tRNA: Methionyl-tRNA
PABP: Poly(A)-binding protein
PIC: 43S pre-initiation complex
rRNAs: Ribosome RNAs

## Introduction

During development many animal cells migrate to form tissues and organs. For example, muscle precursor cells in vertebrates undergo an epithelial-mesenchymal transition (EMT) to delaminate from the somatic dermomyotome and then migrate long distances to construct muscles of the limbs, the diaphragm, and the tongue ( Talbot *et al*., 2019; Vasyutina and Birchmeier, 2006). Neural crest cells also undergo an EMT to detach from the neural tube and travel throughout the embryo to form connective tissue, bones, neurons, and epidermis ( Szabo and Mayor, 2018). During their migrations cells encounter and must invade through extracellular matrices (ECMs) to reach their destinations ( McLennan *et al*., 2020). Cells confront the two main forms of ECM—thin dense laminin and type IV collagen rich basement membranes that surround most tissues and type I and type III collagen rich interstitial matrices that rest between tissues ( Duband and Thiery, 1987; Gros and Tabin, 2014; Pompili *et al*., 2021). In fact, during EMT, cells must immediately breach the underlying epithelial basement membrane to delaminate from the epithelial tissue, and invasive behavior is thought to be a core component of the EMT program ( McClay, 2021). Cell migration and invasion through ECM also plays important roles during immune cell trafficking to sites of infection and injury ( DeDreu *et al*., 2022). Misregulation of invasion underlies many human diseases, such as multiple sclerosis, rheumatoid arthritis, the pregnancy disorder pre-eclampsia, and most notably cancer ( Greter *et al*., 2005; Padmanaban *et al*., 2019; Stanford *et al*., 2016; Zou *et al*., 2019). Understanding how cells migrate and invade through ECM is thus of crucial importance to human development and health.

Invasive cells use specialized F-actin-based plasma membrane protrusions to degrade and breach ECM barriers. These protrusions have been termed podosomes in normal cells and invadopodia in cancer cells ( Cambi and Chavrier, 2021). Collectively these invasive protrusions are referred to as invadosomes, which are likely a related group of membrane-associated protrusive structures that alter their arrangements, dynamics, and composition in response to the environment ( Cambi and Chavrier, 2021; Di Martino *et al*., 2016; Paterson and Courtneidge, 2018). Invadosomes are highly complex and harbor numerous proteins, such as actin regulatory proteins, adhesion proteins, proteases, and signaling molecules that regulate the formation and function of invadosomes in breaking down ECM ( Cambi and Chavrier, 2021; Ezzoukhry *et al*., 2018). Invadosomes are also sites of dynamic vesicle trafficking for protease secretion and membrane addition to support protrusion extension and thus require an expanded membrane trafficking system ( Costa *et al*., 2023; Feng *et al*., 2014; Hastie and Sherwood, 2016; Naegeli *et al*., 2017). To construct and sustain invasive F-actin polymerization, protein secretion, and membrane trafficking, invasive cells also have robust energy acquisition and delivery networks that facilitate dynamic glucose import, glucose processing, mitochondria localization, and oxidative phosphorylation-mediated production of high ATP levels ( Garde *et al*., 2022; Garde and Sherwood, 2021). The building of this complex invasive apparatus requires numerous and diverse genes whose expression is controlled by pro-invasive transcriptional networks ( Medwig-Kinney *et al*., 2020; Pastushenko and Blanpain, 2019). Evidence indicates the translation of the mRNA encoding proteins of the invasive machinery is also regulated to promote cell invasion.

Here, we provide an overview of translation, note aspects of translation that are modulated during invasion, and focus on new studies revealing the roles of ribosome regulation in cell invasion and migration through ECM. Because of the importance of invasion to metastasis, most studies on translation and invasion have been conducted in cancer cell lines and primary tumors in vitro. As cancerous cells are thought to hijack normal invasion mechanisms used in development and homeostasis ( Paterson and Courtneidge, 2018), many of the mechanisms uncovered in tumors will likely apply broadly to other invasive cells.

## Overview of protein translation

**Figure 1.**
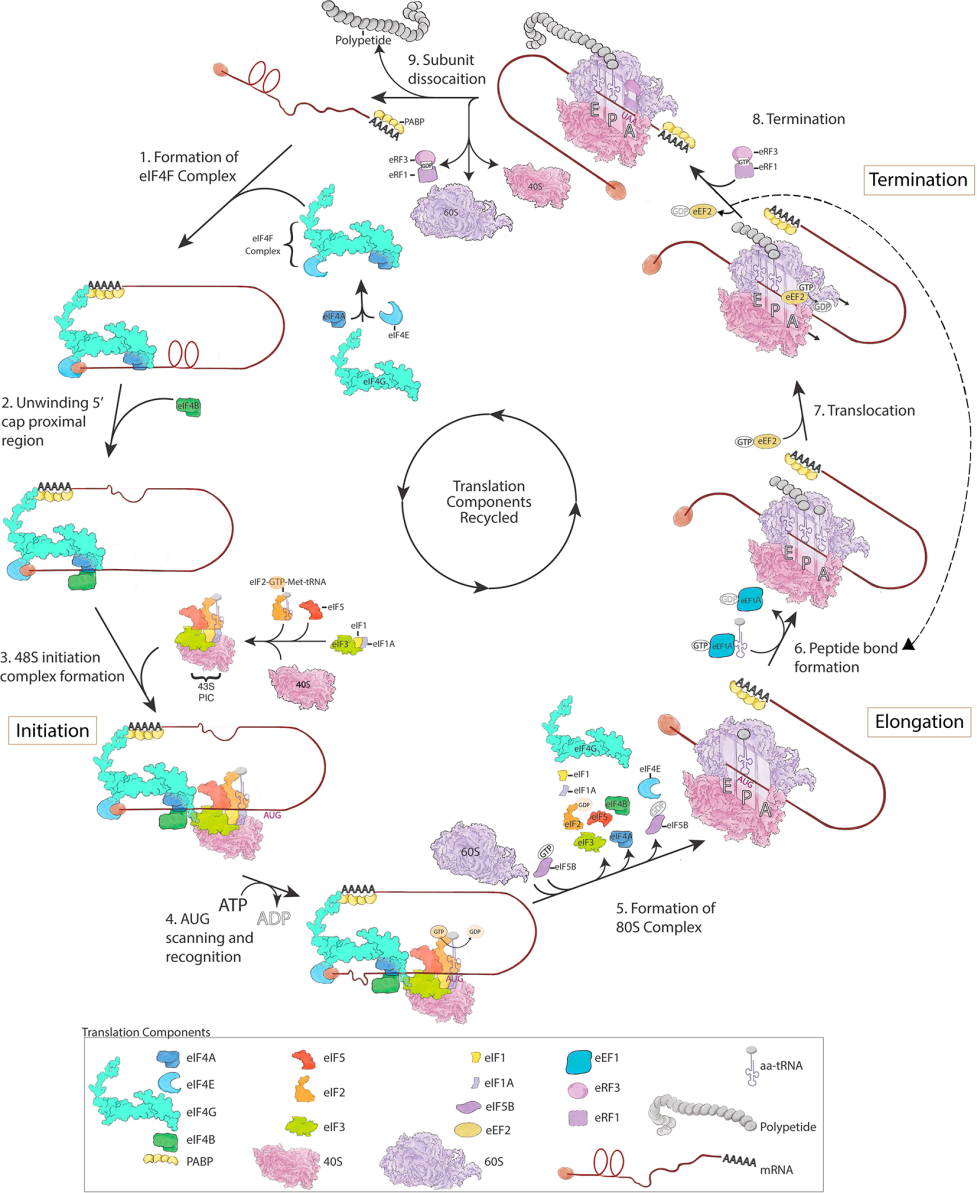
Overview of mRNA Translation. mRNA translation is divided into three steps: (1-5) initiation, (6-7) elongation, and (8-9) termination. For detailed descriptions of these distinct steps that match the figure depiction please see the text. This figure is an original figure produced by the authors for this review article.

Translation is the process by which the information in the unique nucleotide sequence of an mRNA is used to synthesize a distinct protein composed of a chain of amino acids. The information in mRNA is in the form of triplet codes of nucleotides, termed codons, which specifies either a specific amino acid (of a possible 20) or a stop to translation. mRNA is read by ribosomes using aminoacyl-tRNAs (aa-tRNAs) containing anticodons that match with each codon to select and then join specific amino acids together by forming peptide bonds to construct a protein. Translation is terminated by stop codons, which are recognized by release factors (see below). The untranslated sequence at the 5′ end of an mRNA contains a 5′ 7-methylguanosine cap that precedes the start codon, which is the first codon translated. There is also untranslated RNA at the 3′ end of the mRNA that has a 3′ poly(A) tail following the stop codon. Translation is complex and highly regulated and can be separated into three primary phases--initiation, elongation, and termination. Each step is assisted by translation associated factors, and these steps are briefly summarized below ( Figure 1, for a more complete description, see Blanchet and Ranjan (2022) and Hershey *et al*. (2012)).

Cap-dependent translation initiation in eukaryotes occurs when the translation initiation complex (eIF4F), composed of eIF4A, eIF4E and eIF4G, is formed and binds to the 7-methylguanosine cap at the 5′ end of mRNAs ( Smith *et al*., 2021). eIF4E is a cap-binding protein, eIF4G is a scaffolding protein that interacts with poly(A)-binding protein (PABP) at the 3′ end of mRNAs, and eIF4A is a helicase that unwinds secondary structure of mRNA into a single strand in conjunction with the RNA-binding protein eIF4B (or eIF4H, not shown in Figure 1) ( Smith *et al*., 2021). After the eIF4F complex is assembled on an mRNA, it recruits the 43S pre-initiation complex (PIC) through an interaction between eIF4G in the eIF4F complex and eIF3 in the 43S PIC ( Villa *et al*., 2013). The 43S PIC is composed of eIF3, a multiprotein factor comprised of 13 proteins in mammals, and the 40S ribosomal subunit bound to the initiation factors eIF1, eIF1A, eIF5 and the ternary complex (eIF2-GTP-Met-tRNA). The ternary complex harbors the initiator methionyl-tRNA (Met-tRNA) ( Hinnebusch, 2017). The assembly of these components forms the 48S initiation complex ( Figure 1), which scans the mRNA in a 5′ to 3′ direction to locate the start codon (AUG). In addition to cap dependent initiation, in ~10% of mammalian mRNAs, the 40S ribosomal subunit binds directly to an internal region of the mRNA, called the internal ribosome entry site (IRES), which then may proceed to scan the mRNA for the start codon ( Weingarten-Gabbay *et al*., 2016). The recognition of a start AUG codon by the anti-codon of Met-tRNA triggers the hydrolysis of GTP by eIF2 in the ternary complex and allows the 60S ribosomal subunit to join the 48S initiation complex ( Llacer *et al*., 2018; Majumdar and Maitra, 2005; Smith *et al*., 2021). Hydrolysis of GTP by the GTPase eIF2, releases eIF2-GDP from the 48S complex and is thought to lead to the disassembly of many initiation factors ( Blanchet and Ranjan, 2022; Smith *et al*., 2021), although eIF4F might remain at the 5′ cap and allow for recruitment of the next 43S PIC ( Bohlen *et al*., 2020). The assembly of the 60S onto the 48S initiation complex to form the 80S complex is facilitated by GTP-bound eIF5B along with eIF1A. Hydrolysis of GTP by eIF5B and displacement of eIF5B-GDP and eIF1A allows the 80s ribosome to enter the elongation phase of translation ( Pestova *et al*., 2000).

Ribosomes contain three sites where tRNA binds—A (aminoacyl) site that binds newly arriving tRNAs carrying amino acids, P (peptidyl) site where tRNA with the growing polypeptide chain resides, and an E (exit) site, where the tRNA leaves the ribosome after transferring its amino acid. The initiator Met-tRNA, however, functions differently from other tRNAs and is the only tRNA that binds directly to the P site of the ribosome during the translational cycle ( Kolitz and Lorsch, 2010). Subsequent aa-tRNAs are then delivered through GTP-bound eEF1A in a process that involves complementary base pairing between the mRNA codon and the anticodon of the aa-tRNA in the A site of the ribosome ( Figure 1 dashed arrow) ( Knight *et al*., 2020). Codon recognition triggers GTP hydrolysis in eEF1A and eviction of eEF1A-GDP from the A site. In parallel, the ribosome undergoes a conformational change that promotes movement of the aa-tRNA in the A site to contact the tRNA bound to the polypeptide chain in the P site. The movement of the tRNAs from the A to P and P to E sites stimulates ribosome catalyzed peptide bond formation and transfer of the growing polypeptide to the aa-tRNA entering the P site. GTP bound eEF2 then enters the empty A site and through hydrolysis of GTP induces a ribosome conformational change that facilitates movement of the ribosome along the mRNA. The eviction of eEF2-GDP then allows the next aa-tRNA to enter the A site and repeat the elongation cycle ( Knight *et al*., 2020; Taylor *et al*., 2007). Most mRNAs are translated by many ribosomes simultaneously, each following another along the mRNA and forming a polysome.

Upon encountering a stop codon (UAA, UAG or UGA), translation is terminated by two release factors—eRF1, which recognizes stop codons, and eRF3, a GTPase that promotes the activity of eRF1 ( Alkalaeva *et al*., 2006; Zhouravleva *et al*., 1995). In addition to recognizing stop codons, eRF1 also catalyzes peptidyl-tRNA hydrolysis and the release of the newly made protein. Following translation termination, the deacylated tRNA is released and ribosomal subunits disassociate into 40S and 60S subunits (for ribosome disassociation mechanisms see ( Blanchet and Ranjan, 2022)), which are then recycled for use in the translation of other mRNAs ( Shoemaker and Green, 2011).

## Translation regulation and cell invasion

Translation regulators often modulate translation of specific mRNA transcripts by interacting with the unique structural and sequence-specific elements within distinct 5′ or 3′ UTRs of mRNAs ( de la Parra *et al*., 2018; Falletta *et al*., 2017; Ho *et al*., 2021; Smith *et al*., 2021; Song *et al*., 2021; Truitt and Ruggero, 2016). A crucial node for pro-invasive protein regulation is translation initiation ( Verma *et al*., 2019). Alterations in expression of many translation initiation factors and the activity of signaling pathways that act on translation initiation factors are associated with fostering invasion and metastasis in numerous human cancers and in developmental processes ( Chen *et al*., 2019; Esteves *et al*., 2020; Falletta *et al*., 2017; Fan and Guo, 2015; Furic *et al*., 2010; Hao *et al*., 2020; Jaiswal *et al*., 2019; Joyce *et al*., 2017; Martineau *et al*., 2013; Nasr *et al*., 2013; Pinzaglia *et al*., 2015; Ruan *et al*., 2020; Vaysse *et al*., 2015; Wan *et al*., 2015; Wang *et al*., 2016). For example, eIF4G and eIF4E proteins have increased expression across a variety of human cancers and inhibition of eIF4G in prostate cancer cells and eIF4E in ovarian cancer, breast cancer, and melanoma cell lines inhibits invasive activity ( Jaiswal *et al*., 2019; Joyce *et al*., 2017; Nasr *et al*., 2013; Wan *et al*., 2015). Further, experimentally increasing expression of eIF4E in a weakly invasive breast cancer cell line strongly enhances invasive ability ( Nasr *et al*., 2013). Polysome profiling, a method that determines the mRNAs being translated in the cell and the degree of translation of particular mRNAs ( Chasse *et al*., 2017), revealed that translation of MMP9, a metalloproteinase associated with cell invasion, was selectively diminished after reduction of eIF4E ( Nasr *et al*., 2013). Phosphorylation of eIF4E is also elevated in many cancers as a result of RAS/RAF/ERK and PI3K/AKT/mTOR signaling ( Martineau *et al*., 2013) and leads to the preferential increase in translation of mRNAs encoding the pro-invasive matrix metalloproteinase MMP3 and the chemokine CCL2 in prostate cancer cells ( Furic *et al*., 2010).

Other aspects of translational regulation also promote invasion and metastasis in cancer. These include the misexpression of translation elongation factors ( Li *et al*., 2017; Mathews and Hershey, 2015; Xie *et al*., 2018; Zhu *et al*., 2009) and changes in expression and modification of tRNAs that likely favor translation of mRNAs enriched with their cognate codons encoding pro-invasive proteins ( Birch *et al*., 2016; Goodarzi *et al*., 2016; Knight *et al*., 2020; Wan Makhtar *et al*., 2017). Additional translation regulators driving invasion include expression of lncRNAs ( Karakas and Ozpolat, 2021), microRNAs ( Ma *et al*., 2007), N6-methyladenosine (m ^6^A) modification of mRNA transcripts ( Lin *et al*., 2019; Zhao *et al*., 2022), and increased expression of RNA binding proteins that modulate translation ( Evdokimova *et al*., 2009; Wurth *et al*., 2016).

The numerous identified mechanisms regulating mRNA translation that support and drive invasion and migration highlight the importance of modulating translation. Interestingly, recent findings indicate that the ribosome supramolecular complex itself—the molecular machine through which mRNA is turned into proteins—is also regulated to favor invasive behavior.

## Ribosome regulation during cell invasion and migration

### Ribosome biogenesis promotes invasion and migration by expanding translation capacity

Eukaryotic ribosomes are composed of two dissociable subunits called the large and small, referred to as the 60S (47 proteins and the 28S, 5.8S and 5S rRNA molecules) and 40S (33 proteins and 18S rRNA molecule) subunits, which come together on mRNAs to form the 80S ribosome as outlined above ( Ni and Buszczak, 2023a). Ribosome biogenesis involves ribosome assembly within the nucleolus, a nuclear subcompartment, and requires the coordinated production of ribosome RNAs (rRNAs) and ribosomal proteins ( Ni and Buszczak, 2023a). There is now strong evidence indicating that ribosome biogenesis is required within invasive cells to expand translation capacity to produce the numerous cytoskeletal proteins, matrix degrading enzymes, adhesion and signaling proteins, and metabolic enzymes required for invasion and migration.

Studies in PC3 human prostate cancer cells were among the first to implicate ribosome biogenesis in cell invasive behavior. Upregulation of mTOR is strongly associated as a driver of prostate cancer metastasis ( Murugan, 2019). Using polysome profiling in prostate cancer cells, the translation of 144 mRNAs were identified as dependent on mTOR activity ( Hsieh *et al*., 2012). These included 70 mRNAs encoding ribosomal proteins, consistent with the role of mTOR in controlling ribosome biogenesis ( Iadevaia *et al*., 2014), as well as pro-invasive protein encoding mRNAs, such as vimentin, MTA1 (metastasis associated 1), and CD44 ( Hsieh *et al*., 2012). The increased translation of ribosomal proteins alongside pro-invasive proteins suggested a possible role for ribosome biogenesis in cell invasiveness, however, the functional relevance of new ribosome construction in promoting invasion was not examined.

Further support for ribosome biogenesis as a key element in cell invasion was provided by a study investigating EMT. Examination of several mammalian epithelial cell lines and in vivo analysis of neural crest EMT in mice and chick, revealed an upregulation of ribosome biosynthesis during EMT. Evidence included an increase in nascent rRNA synthesis and processing, which initiates ribosome biogenesis ( Gentilella *et al*., 2015), an increase in size of the nucleolus, and increased expression of many proteins that direct ribosome biogenesis ( Prakash *et al*., 2019). Supporting a key role for ribosome biogenesis in cell invasion during EMT, pharmacological inhibition of rRNA synthesis in a mouse epithelial cell line, reduced TGFβ-induced EMT invasion and migration ( Prakash *et al*., 2019). In addition, high levels of RNA Polymerase I, which generates the 5.8S, 18S and 28S rRNAs of the ribosome, are present in invasive breast tumor tissue and pharmacological inhibition of rRNA synthesis in mouse models of breast cancer metastasis (MMTV-PyMT and EO771) reduced metastatic seeding ( Prakash *et al*., 2019).

Recent studies examining *C. elegans* anchor cell invasion have further established a crucial role for ribosome biogenesis in promoting cell invasion and helped clarify a key function for biogenesis in expanding translation capacity to produce the numerous proteins that execute invasion. The anchor cell is a specialized uterine cell that invades through basement membrane separating the uterine and vulval tissues to initiate the uterine-vulval connection during *C. elegans* development ( Kenny-Ganzert and Sherwood, 2023). The anchor cell is born eight hours prior to invasion, grows, and then invades through the basement membrane during a specific 90-minute period using invadosomes armed with protrusive F-actin, microtubules, MMPs, and cell adhesion receptors. By using genome editing to endogenously tag proteins that are required for invasion with genetically encoded fluorophores, it was shown that the levels of these pro-invasive proteins, such as the actin nucleator Arp2/3, microtubule end binding protein EBP-2, integrin adhesion receptor INA-1, glucose importer FGT-1, and small Ras-like GTPase RAP-1, ramp up dramatically approximately two hours prior to invasion and peak in levels at the time of basement membrane breaching ( Costa *et al*., 2023). A recently generated anchor cell transcriptome revealed that mRNAs encoding many ribosomal proteins are enriched in the anchor cell during invasion ( Costa *et al*., 2023). Analysis of ribosomes and ribosome biogenesis markers indicated that a burst of ribosome biogenesis occurs approximately four hours prior to invasion and before the ramp up of pro-invasive protein levels ( Costa *et al*., 2023). Using RNAi at different time points prior to invasion to deplete ribosomal proteins, revealed that only a modest decrease in ribosomal protein levels blocks anchor cell invasion and reduces the translation of many pro-invasive proteins. These results support the notion that early ribosome biogenesis is required to expand translation capacity to produce the many pro-invasive proteins that mediate basement membrane breaching ( Costa *et al*., 2023). Importantly, these observations do not rule out other roles for ribosome biogenesis in supporting invasion, including constructing specialized ribosomes tuned for translating invasive transcripts (see below), as well as ribosomes serving as scaffolds on which signaling components, such as mTOR, can be activated to promote pro-invasive protein production ( Zinzalla *et al*., 2011).

**Figure 2.**
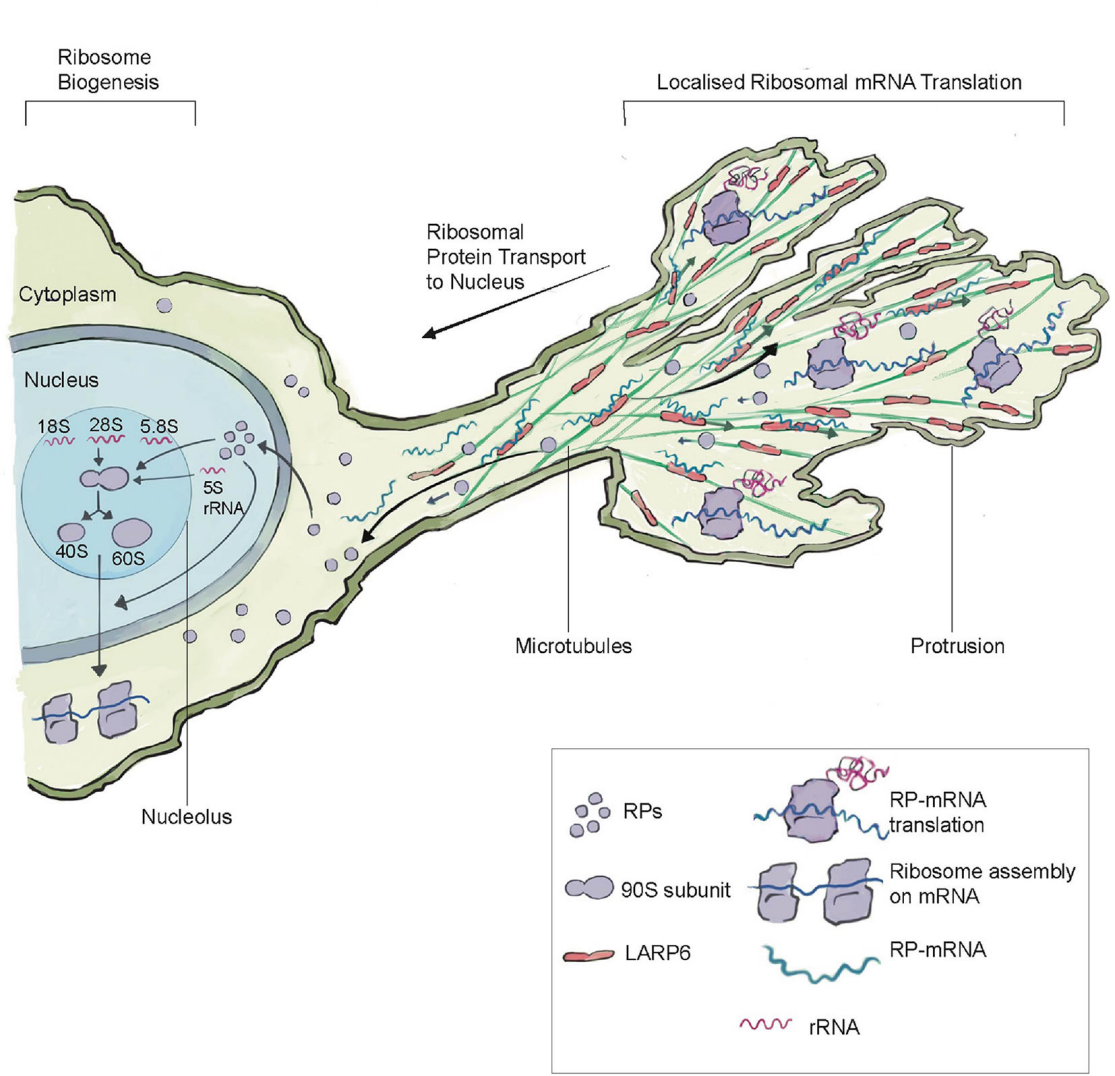
LARP6-mediated Ribosome Biogenesis Regulation in Protrusions. The microtubule binding protein LARP6 enriches in protrusions, where it binds to mRNAs encoding ribosomal protein mRNAs (RP-mRNAs). There is also enrichment of the translation machinery within protrusion, which leads to the translation of ribosomal proteins within protrusions. After translation, ribosomal proteins move back and enter the nucleus, where they are assembled into ribosomes in the nucleolus by combining 5.8S, 5S rRNA, and 46 ribosomal proteins to make the large 60S subunit and the 18S rRNA and 33 ribosomal proteins to make the small 40S subunit. Protrusion formation and LARP6-mediated ribosomal protein translation leads to an increase in ribosome levels. LARP6-mediated ribosome biogenesis may be a form of feed-forward regulation, where migratory and invasive protrusions lead to the production of more translation machinery to support cell invasive and migratory behavior. This figure was adapted from Dermit *et al.* (2020) with permission from Elsevier.

Recent studies have also revealed that many invasive and migratory cells have an elegant system for regulating ribosome biogenesis ( Figure 2). A study examining a diverse group of normal and malignant migratory human cell lines discovered that mRNAs encoding ribosomal proteins are localized to protrusive fronts ( Dermit *et al*., 2020). Localization to protrusions is mediated by LARP6, a microtubule-associated protein that binds to ribosomal protein mRNAs. Protrusive fronts also harbor enrichment of translation initiation and elongation factors ( Figure 2). RiboPuromycylation analysis, an immunofluorescence method to visualize translation sites at the sub-cellular resolution ( Bastide *et al*., 2018), indicated strong colocalization of ribosomal protein-mRNAs and sites of active translation in protrusions ( Dermit *et al*., 2020). Formation of protrusions promoted ribosomal protein translation, enhanced ribosome biogenesis, and boosted the overall protein synthesis of cells. It has been proposed that protrusion regulated ribosome biogenesis might be a form of feedforward control, where the size and stability of protrusions regulates ribosome biogenesis, which further supports migratory and invasive abilities of cells. Notably, the EMT driving transcription factor Snail1, induces both rRNA synthesis to initiate ribosome biogenesis ( Prakash *et al*., 2019) and triggers expression of LARP6 ( Dermit *et al*., 2020), indicating that ribosome biogenesis and its regulation is a critical component the EMT invasion and migratory program.

Together, these studies spanning diverse invasive and migratory cells highlight the importance of ribosome biogenesis in expanding translation capacity to promote invasive activity and cell movement. Further, they have revealed an elegant mechanism of modulating ribosome production involving the mRNA binding protein LARP6, which is a promising therapeutic target to specifically inhibit invasive cells in cancer ( Dermit *et al*., 2020).

### Ribosomes enrich at invadosomes and the endoplasmic reticulum (ER) within invasive cells

**Figure 3.**
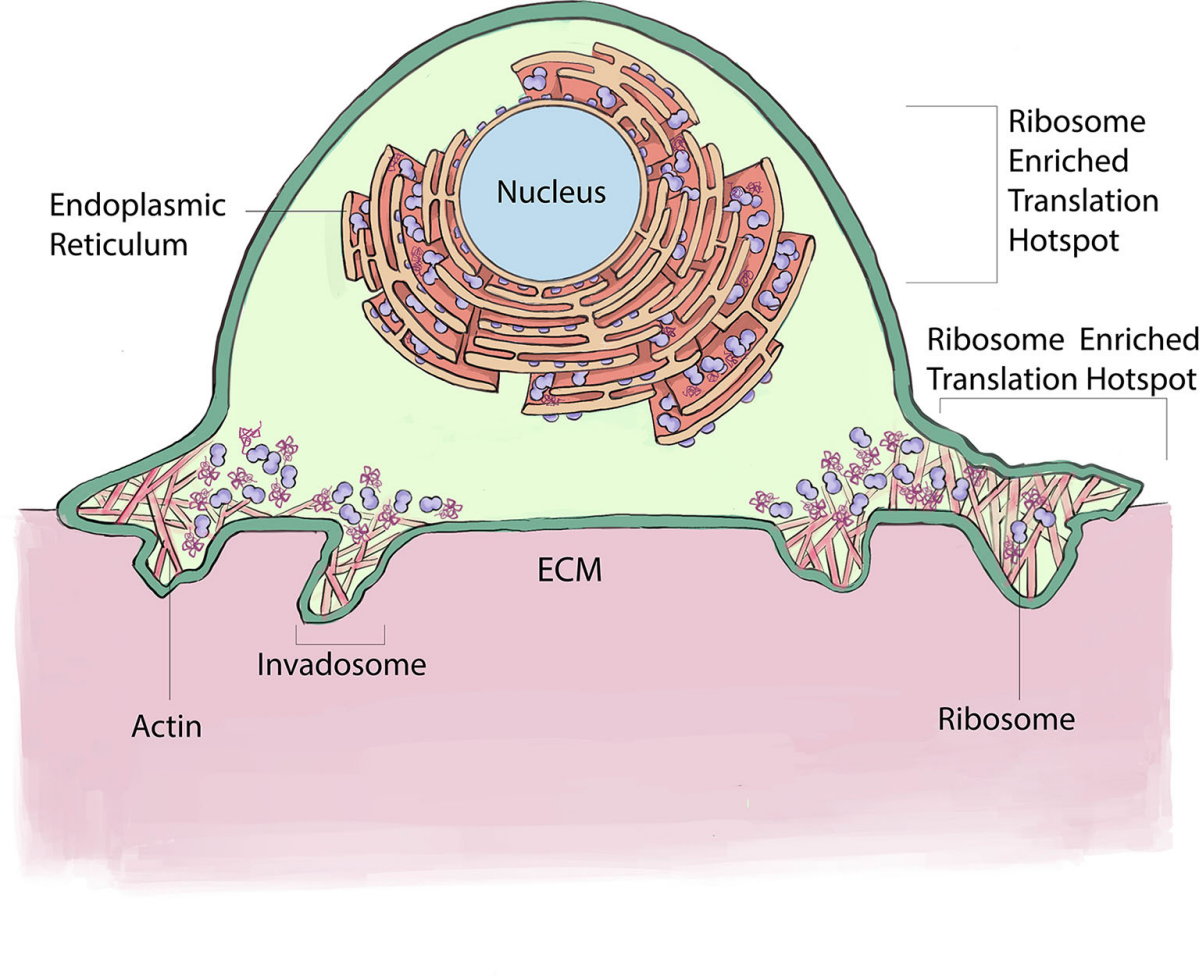
Ribosomes Enrich at the ER and around Invadosomes within invasive cells. Ribosomes enrich at the ER within invasive cells where they support the translation of secretory and transmembrane proteins. In addition, ribosomes localize around ECM degrading invadosomes, where they mediate localized translation necessary for invadosome formation and function. This figure is an original figure produced by the authors for this review article.

Recent advances in ribosome tagging and subcellular biochemical analysis have discovered enrichment of ribosomes and translation at several locations within invasive cells where localized translation mediates important functions ( Figure 3). Using laser capture microdissection, mass spectrometry, immunolocalization, and correlative light and electron microscopy (CLEM) analysis of invadosomes formed in NIH-3T3-Src cells, pioneering studies revealed that ribosomes and translation regulators are highly enriched at these invasive structures ( Ezzoukhry *et al*., 2018). Further, RiboPuromycylation analysis confirmed active translation around F-actin rich invadosomes. Consistent with a potential reliance on this enrichment, targeting of translation regulators with siRNA or treatment with the translation inhibitor ansiomycin, inhibited the ability to form invadosomes and limited invadosome degradation of ECM ( Ezzoukhry *et al*., 2018).

Examining ribosomes in living animals has been challenging because large fluorophores on ribosomal proteins appear to interfere with the complex assembly and tightly packed ribosome structure ( Noma *et al*., 2017). Recently, a split-GFP strategy was developed to label ribosomal proteins endogenously in *C. elegans* ( Costa *et al*., 2023; Noma *et al*., 2017). Examination of endogenous localization of the large ribosomal proteins RPL4 and RPL31 using the split-GFP approach in the invasive anchor cell in *C. elegans*, revealed that approximately three hours prior to invasion, ribosomes co-localize with the endoplasmic reticulum (ER) and Sec61 translocon ( Costa *et al*., 2023). Enrichment at the ER/Sec61 translocon is likely required to translate the many secreted and transmembrane proteins that must enter the endomembrane system to promote invasion. Consistent with high levels of new protein production within the ER, the transcription factor XBP1, the effector of the ER stress sensor IRE1 ( Limia *et al*., 2019), is present at high levels in the nucleus of the anchor cell prior to invasion ( Costa *et al*., 2023). Activated IRE1 directs the splicing of an intron from XBP1 mRNA, which is translated in the cytosol and then trafficked to the nucleus to regulate the expression of genes that counter ER stress ( Cox and Walter, 1996). Interestingly, cytosolic XBP1 localizes to the anchor cell invasive front, suggesting that higher levels of translation are occurring near the site of basement membrane breaching—perhaps to facilitate rapid trafficking through the endomembrane system to the invasive front. Sensitivity to ER stress has been linked to EMT events in vertebrate neural crest cells and several cancers ( Feng *et al*., 2014; Limia *et al*., 2019). Thus, localization of ribosomes to the ER to increase secretory and transmembrane protein production might be a common feature of invasive cells that allows increased translation of proteins that are needed at the cell surface to mediate invasion through ECM.

How ribosomes become enriched in different regions of the cell to support localized translation is unclear. Interestingly, approximately 400 ribosome-associated proteins have been identified in embryonic stem cells from mice ( Simsek *et al*., 2017). Although the functional significance of most of these interactions remain unknown, one of these, pyruvate kinase muscle (PKM), attaches to ER associated ribosomes and appears to bind to, localize, and help translate ER-destined mRNAs ( Simsek *et al*., 2017). It will be important in future studies to determine if PKM or other ribosome-associated proteins help direct ribosomes to the ER and possibly other regions such as invadosomes within invasive cells.

### Ribosomal protein misexpression can drive or inhibit cell invasion in cancer cells

Misexpression of several ribosomal proteins has also been associated with regulating cell invasive activity in cancer. For example, RPL34 is overexpressed in pancreatic cancer and glioma and in vitro cell culture studies have implicated RPL34 in promoting cell invasion through activation of MAPK, p53, and the JAK/STAT3 signaling pathways ( Ji *et al*., 2019; Wei *et al*., 2016). The small ribosomal protein RPS6 and phosphorylated-RPS6 also have increased abundance in many cancers, such as ovarian cancer, esophageal squamous cell carcinoma, and non-small cell lung cancer and promotes invasive activity ( Kim *et al*., 2013; Yang *et al*., 2020). While overexpression of these two ribosomal proteins is strongly associated with cancer progression and cell invasion, it is unclear whether they function within ribosomes to enhance cell invasion or alternatively have pro-invasive activity outside of their roles as components of the ribosome (extra-ribosomal functions).

There is emerging evidence for extra-ribosomal functions of the small ribosomal protein RPS3 and the large ribosome protein RPL3 in regulating cell invasion in cancer cells. RPS3 is misexpressed in multiple cancers ( El Khoury and Nasr, 2021), and overexpression of RPS3 reduces invasiveness of fibrosarcoma cells in vitro ( Kim and Kim, 2006). RPS3 binds to the nucleoside diphosphate kinase A (nm23-H1) ( Kim and Kim, 2006), which acts as a suppressor of metastasis in some tumors ( Yu *et al*., 2021). Binding of RPS3 to nm23-H1 might thus reduce invasiveness by enhancing nm23-H1 function. The large ribosomal protein, RPL3, is also misregulated in cancer and low expression is associated with invasion ( Pecoraro *et al*., 2019). Studies of RPL3 in a human colon cancer cell line indicate that RPL3 binds to the nuclear DNA binding protein poly ADP-ribose polymerase 1 (PARP-1) and represses the transcription of pro-invasive genes ( Pecoraro *et al*., 2019).

The misregulation of several other ribosomal proteins has also been implicated in invasive cancers, such as decreased levels of RPL5 in invasive breast carcinoma ( Fancello *et al*., 2017) and increased expression of RPL19 and RPL31 in malignant prostate cancer and colorectal cancer, respectively ( Bee *et al*., 2006; Sharen *et al*., 2022). Unraveling how misregulated expression of ribosomal proteins alters invasive and migratory activity is challenging as these proteins might have functions within the ribosome, have extra-ribosomal functions, or might activate ribosomal stress pathways ( Kang *et al*., 2021). It is also currently unclear if these identified altered ribosomal protein levels is a normal physiological program that regulates cell invasion or rather an aspect of ribosomal protein misregulation that is only found in certain cancers. Interestingly, RPL31 is upregulated in the anchor cell of *C. elegans* during invasion (see below) ( Costa *et al*., 2023), suggesting that altered expression of ribosomal proteins could be an element of normal cell invasion programs.

### Ribosome heterogeneity and cell invasion and migration

There is abundant evidence that ribosomes can be heterogenous from the assembly of different ribosomal proteins, ribosomal protein paralogs, ribosome-associated factors, post-translational modifications, rRNA variations, and rRNA modifications ( Norris *et al*., 2021). This heterogeneity could provide a cell a remarkable toolkit to create specialized ribosomes to fine tune translation of groups of mRNAs for distinct cell biological functions, including cell migration and invasion. Yet, the functional significance of heterogenous ribosomes remains largely unclear because of the technical challenges of identifying and assessing the function of distinct ribosome populations ( Ni and Buszczak, 2023b).

Two observations are often cited to support the possibility of specialized ribosomes—tissue specific expression of ribosomal proteins and tissue specific phenotypes when different ribosomal protein encoding genes are disrupted ( Norris *et al*., 2021). Importantly, reduced production of ribosomal proteins can lead to a decrease in overall ribosome levels ( Mills and Green, 2017) and translation efficiency of different mRNAs can depend on ribosome levels ( Hu *et al*., 2022; Khajuria *et al*., 2018; Mills and Green, 2017). Thus, tissue specific phenotypes caused by alterations in ribosomal proteins, could simply be caused by tissue specific differences in levels of ribosomes ( Mills and Green, 2017). Further, it is often difficult to rule out extra-ribosomal protein functions, as well as ribosome stress responses caused by ribosome dysfunction ( Mills and Green, 2017). The critical test for specialized ribosomes requires evidence showing that different mRNAs are bound and translated by distinct ribosomes (via ribosome profiling using Ribo-Seq or ART-Seq) and determining if this translation is functionally significant ( Barna *et al*., 2022; Kondrashov *et al*., 2011; Shi *et al*., 2017). While there is emerging support for specialized ribosomes in yeast ( Ferretti *et al*., 2017; Gilbert, 2011; Hu *et al*., 2022), there is only limited evidence in animals ( Ni and Buszczak, 2023b).

The requirement for ribosome biogenesis prior to cell invasive activity and migration provides a potential mechanism for the generation of specialized ribosomes to promote invasion (see above). A transcriptome captured from the *C. elegans* anchor cell at the time of basement membrane invasion revealed upregulation of 13 large ribosomal subunit proteins ( Costa *et al*., 2023). Detailed examination of one of these, RPL31, which might be a regulatory ribosomal protein ( Pech *et al*., 2010), was suggestive of ribosome specialization. First, split-GFP labeling confirmed that RPL31 increased in levels more dramatically leading up to invasion than the core ribosomal protein RPL4 ( Costa *et al*., 2023; Shi *et al*., 2017), which was not significantly upregulated in the transcriptome. Furthermore, RNAi mediated reduction of RPL31 and RPL4 had different effects on translation. Notably, reduction of RLP31 did not decrease translation of the actin regulator Ena/Vasp ( *C. elegans* UNC-34), whereas reduction of RPL4 dramatically decreased Ena/Vasp translation. Further, reduction of RPL31 more severely decreased the translation of the integrin activation GTPase RAP-1 compared to reduction of RPL4 ( Costa *et al*., 2023). This observation supports the idea that RPL31 might be a component of specialized ribosomes that could enhance translation of a group of pro-invasive mRNAs, but not regulate the translation of other proteins. However, these observations do not rule out the possibility that some mRNAs have distinct translation sensitivities to ribosome concentrations that might be differentially affected by RNAi mediated reduction of RPL4 and RPL31 ( Mills and Green, 2017).

It will be important in future studies to perform ribosome profiling in the anchor cell and other invasive cells to examine individual ribosome compositions and the mRNAs ribosomes are actively translating ( Reynoso *et al*., 2015). It will also be crucial to combine this analysis with targeted single cell protein degradation systems such as AID and ZF1 ( Armenti *et al*., 2014; Xiao *et al*., 2023) and overexpression of ribosomal proteins to clarify the possible roles of specialized ribosomes in tuning translation for invasion.

## Outlook

The ability of cells to invade and migrate through tissue and ECM barriers requires the production of many different proteins involved with membrane trafficking, ECM modification, energy production, and the cytoskeleton. Compared with transcription, translational control provides a more direct, rapid, and robust means to adjust protein levels ( Hao *et al*., 2020), which is well suited to meet the dynamic needs of invasive cells to rapidly alter protein levels to overcome and migrate through matrix and tissue barriers. The studies reviewed here highlight the importance and breadth of translational regulation in cell invasion, the challenge of studying translational control, and key gaps in our understanding as well as new opportunities to further our knowledge of how translation regulates cell invasion.

One gap in the field is that it has been dominated by investigation of translation regulation in human cancers and cancer cell lines in vitro. More studies in normal developmental and native physiological settings will help clarify core mechanisms of translational regulation and how these mechanisms are subverted in disease states. An additional challenge is that many aspects of translational regulation are subtle and difficult to disentangle experimentally from critical functions of translation to cellular viability. It will be important in the future to further develop methodologies that separate out core functions of translation from regulatory functions, such as experimental reduction of regulatory components rather than complete loss ( Costa *et al*., 2023; Shi *et al*., 2017), as well as temporal and cell specific removal of translation regulatory components ( Costa *et al*., 2023).

Significant advances have been made in visualizing active translation in cells, imaging ribosomes and translation regulatory components, biochemically isolating ribosomes actively translating mRNA, and revealing the association of ribosomes with many other proteins ( Costa *et al*., 2023; Dermit *et al*., 2020; Shi *et al*., 2017; Simsek *et al*., 2017). It will be vital to build on these techniques and findings to further our understanding of translation in cell invasion and migration. For example, it will be informative to characterize the ~400 protein-ribosome interactions, as they will likely reveal fascinating and unexpected regulation of ribosomes and translation. Furthermore, it will be crucial to expand on the limited number of ribosome composition and profiling studies to perform ribosome profiling in migratory and invasive cells to directly address the functional significance of ribosome heterogeneity in cell invasion. Finally, pushing the frontiers of technological approaches will continue to be important, as super-resolution imaging and expansion microscopy offer the ability to resolve the subcellular localization of individual mRNAs and ribosomes in cells ( Cho and Chang, 2022; Ruland *et al*., 2021), which should lead to deeper insights into ribosome localization and translation. In addition, CRISPR/Cas9 genome engineering is allowing unprecedented manipulation of genomes to probe translation mechanisms, such as the combined genetic removal of all 24 genes encoding paralogs of small ribosomal proteins in a yeast strain ( Hu *et al*., 2022).

Future mechanistic studies on translational regulation in invasive and migratory cells will be critical as they offer to deepen our understanding of the fascinating mechanisms that guide this fundamental cell biological behavior. Studying translation regulation also holds the promise of uncovering new potent therapeutic strategies, such as with the ribosome biogenesis regulator LARP6 ( Dermit *et al*., 2020), to target cell invasive activity more specifically in cancer and immune system disorders.
