# Supplementary material for: Translational regulation of cell invasion through extracellular matrix—an emerging role for ribosomes
Source: F1000Res. 2023 Nov 29;12:1528. [Version 1] doi: 10.12688/f1000research.143519.1 (PMC11019292; doi:10.12688/f1000research.143519.1)
Supplement: Peer review history

# Reviewer report: Reviewer response for version 1

**DOI:** 10.5256/f1000research.157192.r231614

**Published:** 2024-04-15

**Authors:** frederic saltel

**Affiliations:** 1 BRIC BoRdeaux Institute of onCology U1312, INSERM, Rue Léo Saignat, Bordeaux, France

Comments on manuscript

 Title: “ *Translational regulation of cell invasion through extracellular matrix—an emerging role for ribosomes* »

 To Authors,

 This manuscript is very clear and very well written, and it has the advantage of highlighting a new field of investigation. This review perfectly integrates the current problem(s). It is clear that cell invasion through the extracellular matrix is controlled by the regulation of translation.

 There are many examples of this, and it is now necessary to understand and decipher the associated molecular mechanisms, particularly in order to determine whether this pathway has therapeutic potential. In addition, the state of the art is very detailed, making this work accessible to non-specialists in translation and ribosomal proteins.

 After a brief introduction, the authors develop the key elements of translation in a paragraph to make it easier to read and understand the concepts developed in this review. In the following paragraph, the authors cite numerous articles demonstrating that translation regulation regulates invasion, and this in numerous models, particularly in the case of cancers, by highlighting the role of proteins such as eIF4G and eIF4E. In particular, the link between eIF4E and MMP9 is a strong argument.

 The rest of the review focuses on ribosomes, and more specifically on their role as regulators of invasion and migration.

 However, figure 2 needs to be revisited, as the notion of protrusion is not clear and can be applied to many cellular structures, such as lamellipodia, filopodia, etc. In addition, focusing in on this section would make it easier to understand the role of LARP6. Next, the notion of deregulation of ribosomal proteins in this process is well described, as is the notion of heterogeneity of ribosomes formed and involved in the invasion process.

 This concept opens up a very interesting field of research, with the aim of identifying the molecular players that control the interactions between translation and cell invasion. At the same time the second aim is to reveal new therapeutic avenues, given that these mechanisms control the formation of metastases, which remain the leading cause of cancer-related death.

 Minor points :

 A table summarizing the proteins, particularly ribosomal proteins, involved in this process would be an interesting tool for readers.

Is the review written in accessible language?

Yes

Are all factual statements correct and adequately supported by citations?

Yes

Are the conclusions drawn appropriate in the context of the current research literature?

Yes

Is the topic of the review discussed comprehensively in the context of the current literature?

Yes

Reviewer Expertise:

cell biology, invadosome, extracellular matrix, proteomic

I confirm that I have read this submission and believe that I have an appropriate level of expertise to confirm that it is of an acceptable scientific standard, however I have significant reservations, as outlined above.

# Reviewer report: Reviewer response for version 1

**DOI:** 10.5256/f1000research.157192.r248979

**Published:** 2024-03-19

**Authors:** Julie Plastino

**Affiliations:** 1 Ecole Normale Supérieure, Paris, France

This is a nice review of a complex and somewhat understudied subject: how cell invasion is regulated by modifying protein translation.  Much is known about how genes are up or down regulated in cell invasion and cancer, but much less is clear concerning the protein consequences of this regulation.

 Overall, the review does a great job of making a complicated subject with a multitude of molecular players accessible to non-specialists of ribosome biology and protein translation machinery.  There is one small thing that I found confusing: the section “Ribosomal protein misexpression can drive or inhibit cell invasion in cancer cells” seems contradictory with the previous parts, where ribosome enrichment was linked invariably with an increase in invasion presumably by increasing the production of proteins necessary for the increased activity and growth that cancer cells display.  Would it be correct to say that ribosome decrease only drives cancer via activities that aren’t directly connected to protein translation?  Perhaps this can be stated as such, because as it is, this section puts some doubt on what was up until then a strong message that ribosomes and ribosomal function are upregulated for invasion.

 A few “cosmetic” comments:

 1.  In Figure 1, it would be nice to have EPA defined next to its first appearance, so next to the 80S blob between steps 5 and 6.

 2.  Also in Figure 1, a bracket and label for “48S initiation complex” and “80S complex” would be nice (as done for “43S PIC”).

 3.  I think the “the” can be removed from this sentence on page 8: RiboPuromycylation analysis, an immunofluorescence method to visualize translation sites at the sub-cellular resolution (Bastide et al., 2018)…

 4.  In this sentence on page 8, wouldn’t it be better to say “revealed” instead of “discovered”: Recent advances in ribosome tagging and subcellular biochemical analysis have discovered enrichment of ribosomes and translation at several locations within invasive cells where localized translation mediates important functions (Figure 3).

Is the review written in accessible language?

Yes

Are all factual statements correct and adequately supported by citations?

Yes

Are the conclusions drawn appropriate in the context of the current research literature?

Yes

Is the topic of the review discussed comprehensively in the context of the current literature?

Yes

Reviewer Expertise:

actin cytoskeleton, cell motility, C. elegans

I confirm that I have read this submission and believe that I have an appropriate level of expertise to confirm that it is of an acceptable scientific standard.

# Reviewer report: Reviewer response for version 1

**DOI:** 10.5256/f1000research.157192.r244481

**Published:** 2024-03-01

**Authors:** Congying Wu

**Affiliations:** 1 Peking University, Beijing, Beijing, China

The manuscript is constructed in a way that basics of cell translation is first introduced. However, this part reads rather lengthy and key points are buried in lines. I would suggest reorganize this part to provide better readability to the whole text.

 I also suggest the authors to expand on the introduction of cell migration - from a more broad migration view down to the specific type of invading through extracellular migration. mTORC1 is activated at focal adhesions and localized mRNA translation has been suggested to occur at these sites ref [1]and [2]. The differences between matrix-degrading invasion and non-degrading confined migration can also be discussed and relate to ribosomal regulation.  

 As for the second half of the review, the authors focus on ribosomes - its biogenesis, dyregulation of ribosomal proteins and ribosomal heterogeneity. However, the part subtitled “Ribosomal protein misexpression can drive or inhibit cell invasion in cancer cells” is not as well organized as the rest. Logical presentation of evidences and clear enunciation of views are needed.

 Overall, this review is informative and fills the gap between our understanding of the central dogma and cell migration. It will help researchers in cell biology and cancer biology to grasp new directions and will be of great interest to a broad audience.

Is the review written in accessible language?

Yes

Are all factual statements correct and adequately supported by citations?

Yes

Are the conclusions drawn appropriate in the context of the current research literature?

Yes

Is the topic of the review discussed comprehensively in the context of the current literature?

Yes

Reviewer Expertise:

Cell Migration and Adhesion

I confirm that I have read this submission and believe that I have an appropriate level of expertise to confirm that it is of an acceptable scientific standard, however I have significant reservations, as outlined above.

## References

[1] : mTORC1 activity is supported by spatial association with focal adhesions. *J Cell Biol* .2021;220(5) : 10.1083/jcb.202004010 10.1083/jcb.202004010 33635313 PMC7923692

[2] : mTORC1 Mediates Biphasic Mechano-Response to Orchestrate Adhesion-Dependent Cell Growth and Anoikis Resistance. *Adv Sci (Weinh)* .2024;11(6) : 10.1002/advs.202307206 e2307206 10.1002/advs.202307206 38041494 PMC10853740
